# Platelet desialylation is a novel mechanism and a therapeutic target in thrombocytopenia during sepsis: an open-label, multicenter, randomized controlled trial

**DOI:** 10.1186/s13045-017-0476-1

**Published:** 2017-05-11

**Authors:** Mei-feng Li, Xiao-li Li, Kai-liang Fan, Ying-yi Yu, Jing Gong, Shu-ying Geng, Ya-feng Liang, Ling Huang, Ji-hua Qiu, Xing-han Tian, Wen-ting Wang, Xiao-lu Zhang, Qing-xia Yu, Yuan-feng Zhang, Peng Lin, Li-na Wang, Xin Li, Ming Hou, Lu-yi Liu, Jun Peng

**Affiliations:** 1 Department of Hematology, Qilu Hospital, Shandong University, Jinan, 250012 China; 2 Intensive Care Unit, and Clinical Laboratory, Yantai Yuhuangding Hospital Affiliated to Qingdao University, Yantai, 264000 Shandong China; 3 Department of Emergency, Affiliated Hospital of Shandong University of Traditional Chinese Medicine, Jinan, China; 4 Division of Preventive Medicine, Center for Disease Control and Prevention of Yantai Development Zone, Yantai, China; 5 Department of Internal Medicine, Infectious Disease Hospital of Yantai, Yantai, China; 6 Intensive Care Unit, Yantaishan Hospital of Yantai, Yantai, China; 7 Shandong Provincial Key Laboratory of Immunohematology, Qilu Hospital, Shandong University, Jinan, China

**Keywords:** Sepsis, Thrombocytopenia, Oseltamivir, Desialylation, Platelet

## Abstract

**Background:**

Studies in murine models suggested that platelet desialylation was an important mechanism of thrombocytopenia during sepsis.

**Methods:**

First, we performed a prospective, multicenter, observational study that enrolled septic patients with or without thrombocytopenia to determine the association between platelet desialylation and thrombocytopenia in patients with sepsis, severe sepsis, and septic shock. Gender- and age-matched healthy adults were selected as normal controls in analysis of the platelet desialylation levels (study I). Next, we conducted an open-label randomized controlled trial (RCT) in which the patients who had severe sepsis with thrombocytopenia (platelet counts ≤50 × 10^9^/L) were randomly assigned to receive antimicrobial therapy alone (control group) or antimicrobial therapy plus oseltamivir (oseltamivir group) in a 1:1 ratio (study II). The primary outcomes were platelet desialylation level at study entry, overall platelet response rate within 14 days post-randomization, and all-cause mortality within 28 days post-randomization. Secondary outcomes included platelet recovery time, the occurrence of bleeding events, and the amount of platelets transfused within 14 days post-randomization.

**Results:**

The platelet desialylation levels increased significantly in the 127 septic patients with thrombocytopenia compared to the 134 patients without thrombocytopenia. A platelet response was achieved in 45 of the 54 patients in the oseltamivir group (83.3%) compared with 34 of the 52 patients in the control group (65.4%; *P* = 0.045). The median platelet recovery time was 5 days (interquartile range 4–6) in the oseltamivir group compared with 7 days (interquartile range 5–10) in the control group (*P* = 0.003). The amount of platelets transfused decreased significantly in the oseltamivir group compared to the control group (*P* = 0.044). There was no difference in the overall 28-day mortality regardless of whether oseltamivir was used. The Sequential Organ Failure Assessment score and platelet recovery time were independent indicators of oseltamivir therapy. The main reason for all of the mortalities was multiple-organ failure.

**Conclusions:**

Thrombocytopenia was associated with increased platelet desialylation in septic patients. The addition of oseltamivir could significantly increase the platelet response rate, shorten platelet recovery time, and reduce platelet transfusion.

**Trial registration:**

Chinese Clinical Trial Registry, ChiCTR-IPR-16008542.

**Electronic supplementary material:**

The online version of this article (doi:10.1186/s13045-017-0476-1) contains supplementary material, which is available to authorized users.

## Background

Sepsis is a systemic, deleterious host response to infection leading to severe sepsis and possibly septic shock as defined by the Surviving Sepsis Campaign guidelines [1, 2]. Thrombocytopenia is a common finding in sepsis, which is associated with adverse outcomes in patients admitted to the intensive care unit (ICU) [3–5]. In patients with sepsis, thrombocytopenia may not merely be a marker of disease severity but even play a causative role in increased sepsis morbidity and mortality [6, 7]. Thus, the correction of thrombocytopenia becomes a key issue for clinicians during the treatment of sepsis.

The mechanisms of septic thrombocytopenia are not well established and might include bone marrow suppression, immune destruction, disseminated intravascular coagulation (DIC), and platelet chemotaxis induced by inflammatory mediators [8–18]. Grewal et al. found that the marked thrombocytopenia in *Streptococcus pneumonia* sepsis was not mediated by the pathogen itself or the consumption in the process of DIC but due to the Ashwell-Morell receptor (AMR)-dependent clearance of platelets desialylated by NanA neuraminidase [19, 20]. Currently, with the exception of acute platelet transfusion when necessary, there is no effective method to prevent thrombocytopenia.

Sialic acid is a natural sugar acid compound that exists widely across organisms [21]. The ends of glycoprotein chains on platelet membranes are all covered by sialic acid to protect the platelets from being destroyed. Sialidases, also known as neuraminidases, are sialic acid-releasing exoglycosidases that catalyze the removal of terminal sialic acids from sialosides and sialoglycoconjugates in nature [22]. Sialidase widely exists in viruses, bacteria, and mammalian cells [23]. Additionally, resting platelets contain an internal pool of sialidases which are released upon activation from any cause or after a pathogenic bacterium-released new sialidase is introduced into the serum. The endogenous and exogenous sialidases hydrolyze terminal sialic acid moieties from platelet glycoproteins [19, 24]. Several studies in murine models suggested that sialidases released or upregulated during infections hydrolyze sialic acid from platelet glycoproteins [25, 26]. Desialylation leads to the exposure of β-galactose residues on platelets, which can be recognized by AMRs on hepatocytes and eventually results in platelet phagocytosis in the liver [27, 28]. It becomes increasingly evident that desialylation is responsible for, at least in part, the pathogenesis of thrombocytopenia in many diseases and the clearance of transfused platelets after storage [29]. Desialylation-induced platelet removal could possibly be circumvented by adding sialidase inhibitors during sepsis or after refrigeration [19, 30, 31].

Oseltamivir, also known as Tamiflu, is an extensively used and clinically effective anti-influenza drug. It is a viral sialidase inhibitor that prevents the release of progeny virions, thus limiting the spread of infection [32]. Several studies indicate the feasibility that oseltamivir can be used for the treatment of infection-associated thrombocytopenia. Oseltamivir could elevate platelet counts in the treatment of pediatric as well as adult flu patients with thrombocytopenia [30, 33]. Recently, we reported the successful treatment with oseltamivir phosphate of a patient with chronic immune thrombocytopenia (ITP) and influenza [34]. Hence, we hypothesized that thrombocytopenia was associated with increased platelet desialylation in septic patients and that the addition of oseltamivir to an antibiotic therapy would improve the clinical outcome in severe sepsis patients with thrombocytopenia. The study demonstrates that platelet desialylation is a novel mechanism in thrombocytopenia during sepsis. It would provide valuable theoretical foundation and therapeutic targets for the clinical treatment of septic thrombocytopenia.

## Methods

### Study design

**Fig. 1:**
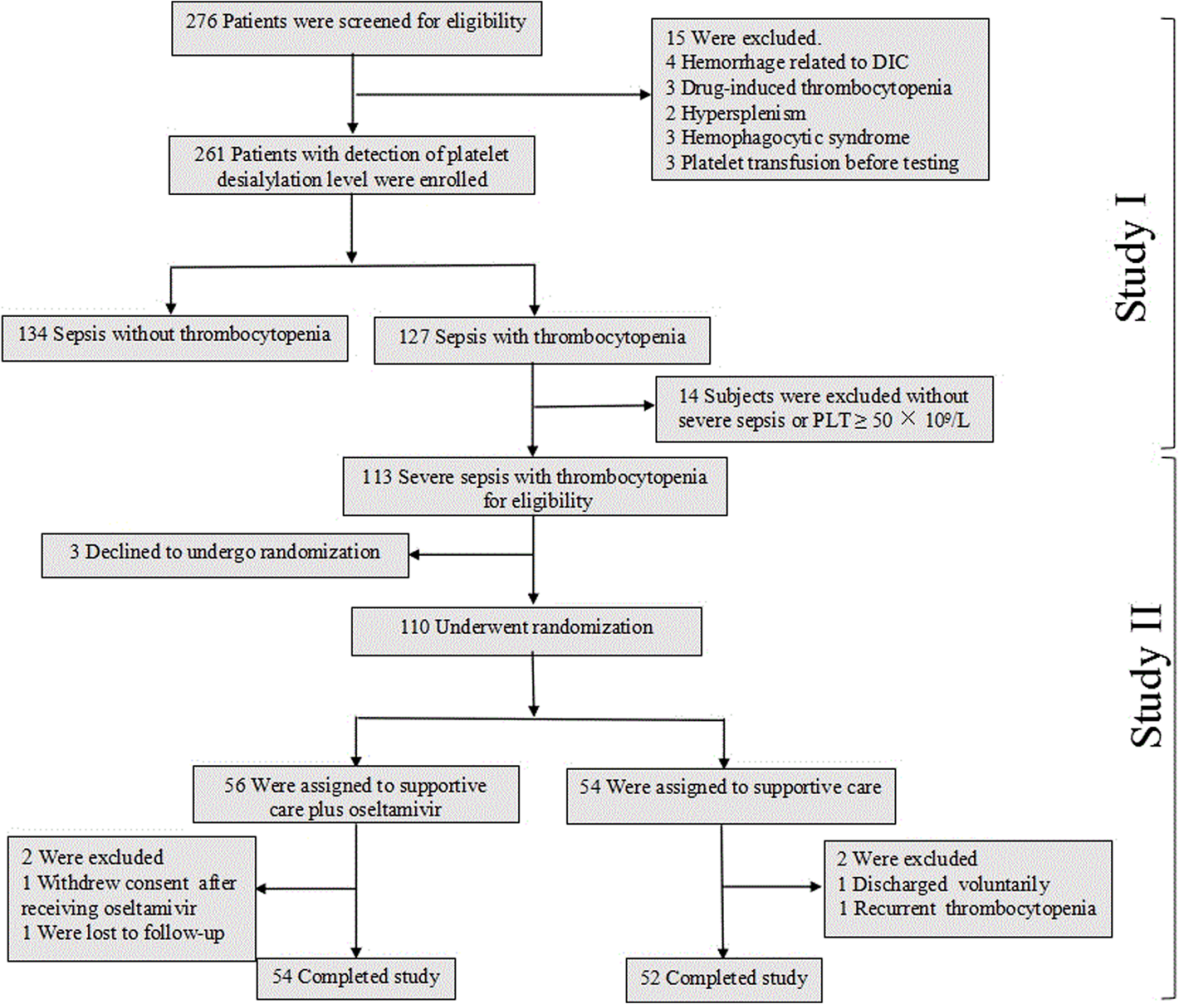
Study flow chart. During the study period, 276 patients were screened for eligibility, and 261 patients met the inclusion criteria. Among them, 127 patients showed thrombocytopenia, of which 113 severe sepsis patients with platelet count less than 50 × 10^9^/L were eligible for the subsequent randomized trial phase. Of them, 110 consented to participate in this phase and were randomly assigned to either receive antimicrobial therapy alone or to receive antimicrobial therapy plus oseltamivir

First, we performed a prospective, multicenter, observational study that enrolled septic patients with or without thrombocytopenia to determine the association between platelet desialylation and thrombocytopenia in patients with sepsis, severe sepsis, and septic shock. Gender- and age-matched healthy adults were selected as normal controls in analysis of the platelet desialylation levels (study I). Next, we conducted an open-label randomized controlled trial (RCT) in which the patients from study I who had severe sepsis with thrombocytopenia (platelet counts ≤50 × 10^9^/L) were randomly assigned to receive antimicrobial therapy alone (control group) or antimicrobial therapy plus oseltamivir (oseltamivir group) in a 1:1 ratio (study II). The study flowchart is shown in Fig. 1.

Both groups received appropriate antimicrobial agents and standard medical support based on the guidelines issued by the Surviving Sepsis Campaign [1]. The oseltamivir group additionally received five full days of oseltamivir therapy. The oseltamivir was administered orally or through a feeding tube at a dose of 75 mg once every 12 h. Time from randomization to the administration of oseltamivir was less than 24 h. The antimicrobial agents were continuously administered until 3 days after the resolution of the physiological abnormalities related to the systemic inflammatory response syndrome (SIRS).

### Study population

The studies were carried out in the Departments of Internal Medicine, and Medical ICUs at Yantai Yuhuangding Hospital, Yantaishan Hospital, Infectious Disease Hospital of Yantai, and Qilu Hospital, Shandong University, Shandong, China, from May 2014 to February 2016.

In study I, patients were eligible for enrollment if they were 18 to 80 years of age; if they presented with sepsis, severe sepsis, or septic shock; and if they had thrombocytopenia (platelet counts less than 100 × 10^9^/L) [35] at the time of admission. To analyze platelet desialylation levels, gender- and age-matched healthy adults were selected as normal controls. In study II, we only included the severe sepsis patients from study I whose platelet counts were less than 50 × 10^9^/L. In agreement with the study of the no inferiority test with a one-sided 5% significance level and a power of 80%, a sample size of 50 patients per group was necessary, given an anticipated dropout rate of 10%.

The diagnostic criteria for sepsis, severe sepsis, and septic shock were based on the Surviving Sepsis Campaign guidelines [1]. Thrombocytopenia was defined as a peripheral blood platelet count less than 100 × 10^9^/L [35]. The exclusion criteria were patients who received chemotherapy or immunosuppressive agents within the prior 6 months, patients with disseminated intravascular coagulation, or drug (e.g., antibiotic or heparin)-associated thrombocytopenia, etc. The detailed eligibility and exclusion criteria are listed in Table 1.

**Table 1:** Eligibility and exclusion criteria of this study

Eligibility criteria	
Study I	1. Patients who were 18 to 80 years of age.
	2. Patients meet the criteria for sepsis which defined by the surviving sepsis campaign.
	2.1 Patients had a documented or suspected infection
	2.2 Patients had some of the following conditions:
	2.2.1 Fever (>38.3 °C) or Hypothermia (<36 °C)
	2.2.2 Heart rate >90 beats/min;
	2.2.3 White blood cell count >12 × 10^9^/L or <4 × 10^9^/L
	2.2.4 Tachypnea
	2.2.5 Altered mental status
	2.2.6 Significant edema or positive fluid balance (>20 mL/kg/24 h)
	2.2.7 Plasma procalcitonin more than two SD above the normal value
	2.2.8 Arterial hypoxemia (PaO_2_/FiO_2_ <300)
	2.2.9 Creatinine increase >44.2 μmol/L
	2.2.10 Arterial hypotension (SBP <90 mmHg, MAP <70 mmHg)
	2.2.11 Coagulation abnormalities (INR >1.5 or APTT >60 s)
	2.2.12 Thrombocytopenia (platelet count <100 × 10^9^/L)
	2.2.13 Hyperbilirubinemia (plasma total bilirubin >70 mmol/L)
	2.2.14 Hyperlactatemia (>1 mmol/L)
	2.2.15 Decreased capillary refill or mottling
	3. Patients whose platelet counts were under 100 × 10^9^/L.
Study II	1. Patients whose platelet counts were under 50 × 10^9^/L.
	2. Patients meet the criteria for severe sepsis which defined by the surviving sepsis campaign.
	2.1 Patients had sepsis-induced tissue hypoperfusion or organ dysfunctionn (any of the following thought to be due to the infection)
	2.1.1 Sepsis-induced hypotension
	2.1.2 Hyperlactatemia (>2 mmol/L)
	2.1.3 Urine output <0.5 mL/kg/h for more than 2 h despite adequate fluid resuscitation
	2.1.4 Acute lung injury with PaO_2_/FiO_2_ <250
	2.1.5 Creatinine >2.0 mg/dL (176.8 mol/L)
	2.1.6 Bilirubin >2 mg/dL (34.2 mol/L)
	2.1.7 Coagulation abnormalities (INR >1.5)
	2.1.8 Thrombocytopenia (platelet count <100 × 10^9^/L)
Exclusion criteria	
1. Patients with malignancies or bone marrow stem cell disorders within the last 2 years	
2. Patients who received chemotherapy or immunosuppressive agents within 6 months	
3. Patients who had a history of ITP or such autoimmune diseases	
4. Patients after cardiopulmonary resuscitation (CPR)	
5. Patients who are less than 18 years or more than 85 years of age	
6. Pregnant or puerperal patients	
7. Patients having acute gastrointestinal hemorrhage	
9. Patients who have a history of bone marrow, lung, liver, kidney, or small bowel transplantation	
10. Patients with end-stage hepatic or renal failure	
11. Patients with disseminated intravascular coagulation (DIC)	
12. Patients with drug (antibiotics or heparin)-associated thrombocytopenia	
13. Gut failure or strict nil by mouth following surgery	

The study was approved by the institutional ethics committee. Written informed consents were obtained from the study participants prior to inclusion in the study. For patients with coma or sedation, the consents were sought from their legal surrogates and were retrospectively gained from those who recovered mental capacity. This study was registered with the Chinese Clinical Trial Registry (ChiCTR; ChiCTR-IPR-16008542).

### Study end points

The primary outcomes were platelet desialylation level at the entry of study I, overall platelet response rate within 14 days post-randomization, and all-cause mortality within 28 days post-randomization. Secondary outcomes included platelet recovery time, the occurrence of bleeding events, and the amount of platelets transfused within 14 days post-randomization.

Platelet response was defined as platelet counts returning to or above a normal level (100 × 10^9^/L).

Platelet recovery time was calculated as the date of randomization to the date when platelet counts were >100 × 10^9^/L.

The criteria for platelet transfusion in patients with severe sepsis were documented as platelets less than 10 × 10^9^/L without significant bleeding or platelets less than 20 × 10^9^/L with significant bleeding risk [36].

### Data collection

For each enrolled patient, the following variables were recorded: (1) general characteristics including age, gender, and primary diseases; (2) severity of illness as assessed by the Acute Physiology and Chronic Health Evaluation (APACHE) II and the Sequential Organ Failure Assessment (SOFA) scores; (3) platelet desialylation level; (4) laboratory data upon admission including hematologic and chemistry tests, blood coagulation assays, and arterial blood gas analysis; (5) screening tests for virus infection, serum galactomannan and (1 → 3)-β-d-glucan assays, and bacterial culture from clinical specimens such as sputum, blood, urine, secretions, and drainage fluid; (6) interventions including mechanical ventilation and continuous renal replacement therapy (CRRT); (7) the development of complications such as acute respiratory distress syndrome (ARDS), DIC, and bleeding events; and (8) the amount of platelets transfused. Time from sepsis onset for measurement of all laboratory parameters was limited within 24 h.

### Assessment of platelet desialylation

Ethylenediaminetetraacetic acid anti-coagulated whole blood was obtained from sepsis patients and healthy controls by venipuncture. Platelet-rich plasma was isolated by centrifugation at 200 g for 10 min. Fresh platelets were separated from platelet-rich plasma by centrifugation at 850*g* for 5 min, washed in buffer A (140 mM NaCl, 5 mM KCl, 12 mM trisodium citrate, 10 mM glucose, 12.5 mM sucrose, 1 μg/mL prostaglandin E1, pH 6.0), and resuspended in buffer B (10 mM HEPES, 140 mM NaCl, 3 mM KCl, 0.5 mM MgCl_2_, 10 mM glucose, and 0.5 mM NaHCO_3_, pH 7.4).

To detect platelet desialylation, PE-Cy5-labeled anti-human CD41 monoclonal antibodies (20 μL per test, 4 μg/mL; BD Bioscience, San Jose, CA, USA) were used to label human platelets. FITC-labeled *Ricinus communis* agglutinin I (RCA-I, 5 μg/mL; Vector Laboratories, Burlingame, CA, USA), *Erythrina cristagalli* lectin (ECL, 10 μg/mL; EY Laboratories, San Mateo, CA, USA), and Succinyl *Triticum vulgare* lectin (sWGA, 0.1 μg/mL; EY Laboratories) were used to analyze sialic acid exposure on platelet surfaces. Platelets (1 × 10^6^) were incubated with anti-CD41 and RCA-I, ECL, or sWGA, respectively for 20 min, washed twice, and resuspended. The percentages of platelets positive for RCA-I, ECL, or sWGA represented the levels of platelet desialylation [31].

### Statistics

Data analysis was performed using the SPSS 16.0 statistical software package (SPSS Inc., Chicago, IL, USA). Normally distributed variables were expressed as the mean ± standard deviation (SD), while skewed variables were expressed as the median (interquartile range). Between-group comparisons were evaluated by the two-tailed unpaired Student’s *t* test (for normally distributed data) and Mann-Whitney *U* test (for skewed data). The comparison of categorical variables was processed by the chi-squared (*χ* ^2^) test. Multiple stepwise regression analysis was used to assess the association between platelet count and the other variables, while independent variables correlating with the 28-day mortality were determined by a multiple logistic regression analysis. Survival analysis was conducted based on whether oseltamivir was used, and differences in survival rates between groups were compared using the log-rank test. A multivariate analysis with the Cox proportional hazards regression model was used to assess the influence of each variable on the response to oseltamivir treatment. A *p* value less than 0.05 was considered statistically significant.

## Results

### Baseline characteristics and clinical features for all of the patients

During the study period, 276 patients were admitted, and 261 patients met the criteria of study I (Fig. 1). Among them, 134 were septic patients without thrombocytopenia, and 127 were patients with thrombocytopenia. Additional file 1: Table S2 shows the characteristics of the 261 patients upon admission.

Among the 127 septic patients with thrombocytopenia, 110 severe sepsis patients whose platelet counts were less than 50 × 10^9^/L were enrolled in study II. After randomization, 52 patients were treated with antibiotics alone (the control group), whereas the other 54 patients were treated with antibiotics plus oseltamivir (the oseltamivir group). The 28-day follow-up was completed in 96.4% of the patients (Fig. 1).

### Comparison of septic patients with or without thrombocytopenia

Table 2 details the demographic data of septic patients with or without thrombocytopenia. Patients with thrombocytopenia had higher SOFA scores [10 (5, 12) vs 5 (3.25, 7), *P* < 0.001], PCT levels [6.69 (1.0, 38.9) vs 1.55 (0.32, 5.36), *P* < 0.001], and creatinine levels [88 (63.3, 225) vs 81 (56, 119.0) μmol/L, *P* = 0.036] but had a lower mean arterial pressure (79.6 ± 14.7 vs 86.8 ± 15.5 mmHg, *P* < 0.001) than patients without thrombocytopenia. Patients with thrombocytopenia had a higher occurrence of renal replacement (25.2 vs 14.2%, *P* = 0.029) and septic shock (18.9 vs 8.2%, *P* = 0.017) than patients without thrombocytopenia. The 28-day mortality rate was higher in patients with thrombocytopenia than in patients without thrombocytopenia (36.2 vs 14.2%, respectively; *P* < 0.001). And there were significant differences between patients with and without thrombocytopenia in the incidence rate of bloodstream infection, lung infection, and the infection pathogens of *Escherichia coli*, New Bunya virus, *Pseudomonas aeruginosa*, and *Staphylococcus aureus*. Multiple stepwise regression analysis of the association of thrombocytopenia and the clinical parameters with significant differences in Table 2 revealed that the bloodstream infection and the infection pathogen of New Bunya virus were independent risk factors for thrombocytopenia in sepsis patients. In contrast, the infection caused by *P. aeruginosa* and *S. aureus* were negatively associated with thrombocytopenia (Additional file 1: Table S2). Multivariate logistic regression analyses were performed to determine if there were any associations between risk factors and 28-day mortality in the 261 patients, the risk factors including age, gender, SOFA, platelet count, mechanical ventilation, continuous renal replacement therapy (CRRT), acute respiratory distress syndrome (ARDS), and septic shock (Additional file 1: Table S3). It was demonstrated that platelet count was an independent factor negatively associated with the 28-day mortality rate (OR = 0.963, 95% CI 0.930–0.997, *P* = 0.033), which was consistent with other reports [3]. SOFA was also an independent risk factor for 28-day mortality rate (OR = 1.808, 95% CI 1.246–2.623, *P* = 0.002).

**Table 2:** Comparison between the sepsis patients with and without thrombocytopenia

Variables	Sepsis without thrombocytopenia (*n* = 134)	Sepsis with thrombocytopenia (*n* = 127)	*P* value
Age (year)	69.5 (54.5, 78.3)	68 (55.0, 77.0)	0.468
Male	91 (67.9)	84 (66.1)	0.793
APACHE II	18.5 (15.3, 23.8)	20 (10, 24.8)	0.861
Predicted death rate (%)	31.5 (21, 45.2)	37.3 (20.4, 53.5)	0.217
SOFA	5 (3.25, 7)	10 (5, 12)	<0.001
PCT (ng/mL)	1.55 (0.32, 5.36)	6.69 (1.0, 38.9)	<0.001
SA (mmol/L)	732.8 ± 156.5	669.1 ± 143.9	0.089
MAP (mmHg)	86.8 ± 15.5	79.6 ± 14.7	<0.001
Heart rate (beat/min)	93.3 (88.0, 106.3)	98 (84, 116)	0.221
White blood cell (×10^9^/L)	12.5 (8.4, 15.4)	14.1 (7.81, 15.5)	0.352
Platelet count (×10^9^/L)	196.5 (143.5, 299.75)	45 (33.25, 64.75)	<0.001
BUN (mmol/L)	7.4 (5.18, 17.3)	7.51 (4.06, 14.6)	0.402
Creatinine (μmol/L)	81 (56.0, 119.0)	88 (63.3, 225.0)	0.036
Total bilirubin (μmol/L)	14.8 (9.9, 23.1)	18.8 (11.4, 35.0)	0.098
Albumin (g/L)	25.5 ± 3.48	24.8 ± 5.75	0.267
Infection pathogens (%)			
*Escherichia coli*	12 (8.96)	25 (19.7)	0.020
*Klebsiella pneumoniae*	22 (16.4)	11 (8.66)	0.065
*Pseudomonas aeruginosa*	30 (22.4)	11 (8.66)	0.030
*Acinetobacter baumanii*	13 (9.70)	7 (5.51)	0.248
*Staphylococcus aureus*	21 (15.7)	5 (3.93)	0.020
*Streptococcus pneumonae*	1 (0.75)	2 (1.57)	0.641
*Enterococcus faecium*	5 (3.73)	4 (3.15)	0.999
Chicken *enterococcus*	1 (0.75)	2 (1.57)	0.614
*Proteus mirabilis*	0	1 (0.78)	0.487
*Candida albicans*	1 (0.75)	2 (1.57)	0.614
*Aspergillus*	3 (2.24)	7 (5.51)	0.207
Viruses	17 (12.7)	36 (28.3)	0.002
New Bunya virus	0	21 (16.5)	<0.001
Influenza A virus subtype H1N1	6 (4.48)	6 (4.72)	0.999
Others	11 (10.4)	9 (9.45)	0.818
Infection sites (%)			
Lung	69 (51.5)	29 (22.8)	<0.001
Bloodstream	8 (5.97)	33 (23.6)	<0.001
Abdominal cavity	11 (8.21)	15 (11.1)	0.409
Hepatobiliary system	8 (5.97)	9 (6.9)	0.804
Urinary system	6 (4.48)	8 (6.9)	0.589
Surgical incision	3 (2.24)	3 (6.9)	0.632
Skin and soft tissue	2 (1.49)	2 (1.4)	0.668
Central nervous system	2 (1.49)	1 (1.4)	0.520
Others	25 (18.7)	27 (21.3)	0.644
Platelet desialylation level (%)			
RCA-I	90.14 ± 8.88	95.64 ± 4.19	0.014
ECL	2.33 ± 1.90	5.70 ± 4.37	<0.001
sWGA	0.15 ± 0.95	0.50 ± 0.293	<0.001
Mechanical ventilation	63 (47.0)	72 (56.7)	0.137
Renal replacement therapy	19 (14.2)	32 (25.2)	0.029
Septic shock	11 (8.2)	24 (18.9)	0.017
ARDS	30 (22.4)	34 (26.8)	0.472
Bleeding	32 (23.9)	29 (22.8)	0.884
28-day mortality	19 (14.2)	46 (36.2)	<0.001

Data are presented as number (percentage) or median (interquartile range); *APACHE II* acute physiology and chronic health evaluation scoring system, *SOFA* sequential organ failure assessment, *PCT* procalcitonin, *ARDS* acute respiratory distress syndrome, *MAP* mean arterial pressure, *SA* sialic acid, *RCA-I Ricinus communis* agglutinin I, *ECL Erythrina cristagalli* lectin, *sWGA* Succinyl *Triticum vulgare* lectin, *BUN* blood urea nitrogen

To compare the platelet desialylation levels between septic patients with or without thrombocytopenia, we quantified the binding of fluorescein-conjugated lectins, which target exposed sialic acids following GP desialylation as follows: ECL, which has a specificity toward galactose residues and the highest binding activity toward galactosyl (β-1,4) *N*-acetylglucosamine; RCA-I, which binds preferentially to oligosaccharides ending in galactose but may also interact with *N*-acetylgalactosamine; and sWGA, which has a specificity toward *N*-acetylglucosamine. The data demonstrated that platelet desialylation increased significantly in septic patients with thrombocytopenia compared to those without thrombocytopenia, as detected with ECL, RCA-I, or sWGA lectins (Additional file 1: Figure S1).

### Comparison of antimicrobial therapy plus oseltamivir with antimicrobial therapy alone in severe sepsis patients with thrombocytopenia

The baseline demographic data of patients treated with antibiotics plus oseltamivir (oseltamivir group) or with antibiotics alone (control group) are listed in Additional file 1: Table S4. There were no differences between the two study groups (at a significance level of less than 0.05) with respect to any demographic variable, showing a comparability of our data. There was no difference in adequate antibiotic treatment between groups. The duration of the antimicrobial treatment was not different in the oseltamivir group compared with the control group [11 (9, 13.25) vs 10 (9, 13); *P* = 0.418]. No significant between-group differences were found with respect to the sites of infection or the infectious pathogens. All of the laboratory tests presented no significant differences between the two groups, except for some isolated indices.

Table 3 demonstrates the responses and outcomes of patients in the oseltamivir group and the control group. Within 15 days after randomization, platelet response was achieved in 45 of the 54 patients in the oseltamivir group (83.3%) compared with 34 of the 52 patients in the control group (65.4%; *P* = 0.045). In patients who had a platelet response, the platelet recovery time was 5 days with quartiles of 4–6 days in the oseltamivir group compared with 7 days with quartiles of 5–10 days in the control group (*P* = 0.003). Additional file 1: Figure S2 displays the changes in the platelet counts of the oseltamivir and control groups over time. At baseline, the platelet counts were 41.81 ± 10.29 × 10^9^/L in the oseltamivir group and 46.69 ± 9.13 × 10^9^/L in the control group, with no significant difference between these two groups (*P* = 0.560). Platelet counts in both groups increased over time and reached 91.30 ± 21.51 × 10^9^/L in the oseltamivir group and 58.17 ± 32.99 × 10^9^/L in the control group on the fourth day after the initiation of oseltamivir treatment (*P* = 0.031). From day 4 through day 9, the difference continued to be statistically significant between the groups, and on the 10th day, platelet counts were 198.8 ± 82.6 × 10^9^/L in the oseltamivir group and 147.7 ± 81.78 × 10^9^/L in the control group (*P* = 0.684). The amount of platelets transfused decreased significantly in the oseltamivir group (0.676 ± 1.36 apheresis units) compared to the control group (1.35 ± 1.98 apheresis units; *P* = 0.044; Table 3). The data indicated that oseltamivir could shorten the platelet recovery time and reduce platelet transfusion quantity. No special adverse events during the oseltamivir therapy were observed (Additional file 1: Table S5). The platelet desialylation levels were analyzed in 25 patients after oseltamivir therapy. The sWGA FITC-labeled lectins declined significantly compared to those before treatment. However, neither RCA-I nor ECL FITC-labeled lectins showed significant change after oseltamivir treatment (Additional file 1: Figure S4).

**Table 3:** Responses and outcomes of patients in the oseltamivir group and control group

Variables	Control (*n* = 52)	Oseltamivir (*n* = 54)	*P* value
Platelet response rate, % (*n*)	65.4 (34)	83.3 (45)	0.045
Platelet recovery time (day)	7 (5, 10)	5 (4, 6)	0.003
Platelet transfusion (apheresis unit)	1.35 ± 1.98	0.676 ± 1.36	0.044
Bleeding (%)	9 (17.3)	11 (20.4)	0.805
28-day mortality (%)	22 (42.3)	19 (35.2)	0.550

The overall 28-day mortality rate in the two groups combined was 38.7%, with 35.2% in the oseltamivir group and 42.3% in the control group (*P* = 0.55; Table 3). There was no difference in survival over time based on whether oseltamivir was used (Additional file 1: Figure S3). Multivariate Cox proportional hazards models were used to estimate the variables that were associated with the responses in the oseltamivir and control groups, respectively, including age, sex, APACHE II score, SOFA score, platelet recovery time, and platelet count. It was revealed that the SOFA score and platelet recovery time were independent indicators of oseltamivir therapy (Table 4). The main reason for all of the mortalities was multiple-organ failure.

**Table 4:** Multivariate Cox proportional hazard analysis of oseltamivir therapy

Variables	Hazard ratio	95% CI		*P* value
		Lower	Upper	
Age	1.020	0.990	1.051	0.189
Sex	0.938	0.726	1.212	0.626
APACHE II	1.105	0.951	1.284	0.191
SOFA	0.578	0.365	0.965	0.029
Platelet recovery time	0.798	0.636	0.990	0.042
Platelet count	1.011	0.983	1.039	0.463

*95% CI* indicates 95% confidence interval. APACHE II and SOFA score were obtained at patients’ admission. *APACHE II* acute physiology and chronic health evaluation scoring system, *SOFA* sequential organ failure assessment

Oseltamivir treatment was well-tolerated in our study. No patient reported side effects that were severe enough to necessitate the discontinuation of treatment. Eighteen patients with the digestive tract and respiratory diseases or both tolerated the medication well (Additional file 1: Table S5). On the whole, the incidence of adverse events in oseltamivir group was similar to the control group.

## Discussion

According to the 2016 international consensus definitions for sepsis and septic shock (Sepsis-3), sepsis is defined as life-threatening organ dysfunction caused by a dysregulated host response to an infection. Organ dysfunction can be identified as an acute change in the total SOFA score ≥2 consequent to the infection. The new definition of sepsis reflects an up-to-date view of pathobiology, particularly in regards to what distinguishes sepsis from uncomplicated infection [37]. Although our study was initiated in 2014, at which point the diagnostic criteria for sepsis was based on the Surviving Sepsis Campaign guidelines (Sepsis-2), 253 of the 261 sepsis patients included had SOFA scores ≥2. Thus, even by the more “strict” criteria of Sepsis-3, most patients had sepsis involving organ dysfunction rather than an infection plus an accompanying inflammatory response alone.

In this study, we demonstrated that the platelet desialylation increased significantly in septic patients with thrombocytopenia compared to those without thrombocytopenia as detected with ECL, RCA-I, or sWGA lectins, indicating that thrombocytopenia was associated with increased platelet desialylation in septic patients. This research confirms the desialylation pathogenesis of sepsis thrombocytopenia and provides new countermeasures for the clinical treatment of thrombocytopenia in sepsis patients.

Greenberg et al. pretreated platelets with neuraminidases in vitro and found that removal of 15% of platelet sialic acid led to complete platelet clearance within 1 h after injection back to the circulation [38]. Moreover, an exponential correlation was showed between the shortening of platelet life span in the circulation and the content of sialic acid removed by neuraminidases in vitro [39]. In addition, direct injection of neuraminidases to mammals markedly accelerated the clearance and shorted the life span of platelets [40, 41]. These studies suggest that neuraminidases can significantly affect platelet life span by causing platelet desialylation.

Next, we performed a randomized controlled study in which the severe sepsis patients who had thrombocytopenia with platelet counts less than 50 × 10^9^/L were randomly assigned to receive antimicrobial therapy alone or antimicrobial therapy plus oseltamivir. The data demonstrated that the addition of oseltamivir to antibiotic therapy could significantly increase the platelet response rate, shorten the platelet recovery time, and reduce platelet transfusion. The oseltamivir was administered for 5 days according to the drug instruction, yet its effect appeared to persist well beyond discontinuation of the drug and the difference continued to be statistically significant between the oseltamivir and control group.

It is not clear whether correcting the platelet count will result in a decrease in mortality. In this study, the multivariate analysis by Cox proportional hazards models showed that the SOFA score and platelet recovery time were independent indicators of oseltamivir therapy. Therefore, the main cause for all of the cases of mortality was multiple-organ failure [42]. This result might explain why there was no difference in the overall 28-day mortality rate or in the survival over time based on whether oseltamivir was used in this study.

However, in critical care medicine, it is commonly agreeable that when evaluating an intervention in critically ill patients, it is not necessarily associated with improved mortality. In a study which systematically reviewed the available literature to identify multicenter RCTs assessing mortality as a primary outcome in ICU patients, it is demonstrated that relatively few of the RCTs conducted in ICUs and using mortality as a primary outcome show a beneficial impact of the intervention on the survival of critically ill patients [43]. Many other studies also stimulate research on end points other than crude differences in mortality rates [44].

One limitation of our trial is its open-label nature. Given that most severe sepsis patients with thrombocytopenia in study II are acute and/or critically ill patients with multiple-organ failure, a blinded study do not seem possible. Moreover, all end points in our study were based on objectively measured laboratory values rather than clinical impressions or assessments of symptoms which would be impacted by a clinician’s subjective bias during data collection and evaluation of study parameters. So a placebo with blinding was not included in the study design.

Septic patients with thrombocytopenia carry particularly poor prognosis and often require platelet transfusions. Platelets are a limited resource and have potential for causing transfusion reactions. Beyond the role in hemostasis and thrombosis, platelets modulate inflammatory reactions and immune responses [45]. The study showed that despite the absence of a difference in 28-day mortality, oseltamivir shortened the recovery time from thrombocytopenia, reduced the need for platelet transfusion, and saved the cost. Therefore, a medication with a relatively good safety profile which is readily available and can decrease the requirements for platelet transfusions would be beneficial. Additionally, early recovery from thrombocytopenia helps to prevent the coagulopathy and enhance the immunity. Our study confirmed that desialylation pathogenesis of sepsis thrombocytopenia provides new strategy for the clinical treatment of thrombocytopenia in sepsis patients.

## Conclusions

Thrombocytopenia was associated with increased platelet desialylation in septic patients. The addition of oseltamivir could significantly increase the platelet response rate, shorten the platelet recovery time and reduce platelet transfusion.

## Additional files

Additional file 1: **Figure S1.** Flow cytometric analysis of β-galactose or β-GlcNAc exposure on platelet glycoproteins. **Figure S2.** Changes in platelet counts of the oseltamivir and control groups over time. **Figure S3.** Survival curves of thrombocytopenia patients, according to treatment group. **Figure S4.** Platelet desialylation levels before and after the oseltamivir treatment. **Table S1.** Baseline characteristics of the all patients (*n* = 261). **Table S2.** Factors affecting the thrombocytopenia in the multiple stepwise regression model (*n* = 261). **Table S3.** Multivariate analysis of the effects of various clinical parameters at study entry on the odds ratio for 28-day mortality (*n* = 261). **Table S4.** Baseline demographic data of patients treated with antibiotics plus oseltamivir (oseltamivir group) or with antibiotics alone (control group). **Table S5.** The adverse events in oseltamivir treatment group and control group in study II (*n* = 106). (DOC 1586 kb)
