# Supplementary material for: Platelet desialylation is a novel mechanism and a therapeutic target in thrombocytopenia during sepsis: an open-label, multicenter, randomized controlled trial
Source: J Hematol Oncol. 2017 May 11;10:104. doi: 10.1186/s13045-017-0476-1 (PMC5426054; doi:10.1186/s13045-017-0476-1)
Supplement: Supplementary file 1 — Figure S1. Flow cytometric analysis of β-galactose or β-GlcNAc exposure on platelet glycoproteins. Figure S2. Changes in platelet counts of the oseltamivir and control groups over time. Figure S3. Survival curves of thrombocytopenia patients, according to treatment group. Figure S4. Platelet desialylation levels before and after the oseltamivir treatment. Table S1. Baseline characteristics of the all patients (n = 261). Table S2. Factors affecting the thrombocytopenia in the multiple stepwise regression model (n = 261). Table S3. Multivariate analysis of the effects of various clinical parameters at study entry on the odds ratio for 28-day mortality (n = 261). Table S4. Baseline demographic data of patients treated with antibiotics plus oseltamivir (oseltamivir group) or with antibiotics alone (control group). Table S5. The adverse events in oseltamivir treatment group and control group in study II (n = 106). (DOC 1586 kb) [file 13045_2017_476_MOESM1_ESM.doc]

**Additional file 1**

**Figures**


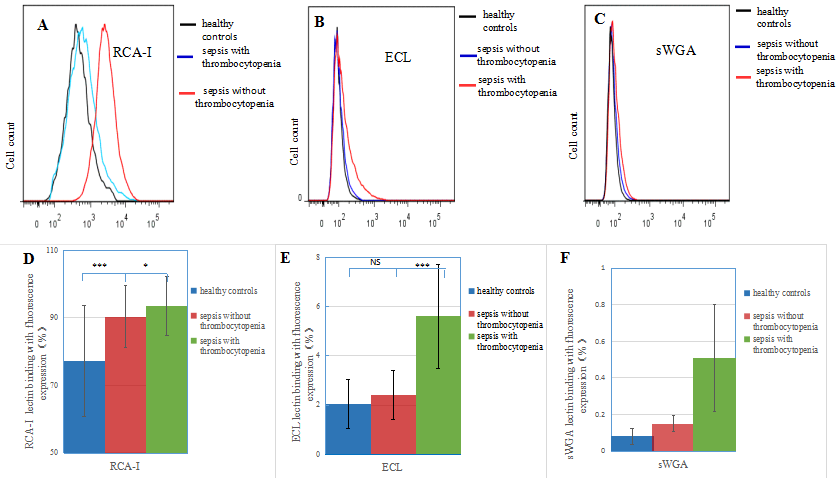


**Figure S1. Flow cytometric analysis of β-galactose or β-GlcNAc exposure on platelet glycoproteins.** (A-C) Representative pictures of flow cytometric analysis of β-galactose or β-GlcNAc exposure on platelet glycoproteinsas, as detected with RCA-I (A), ECL (B) or sWGA (C) FITC-labeled lectins. (D-F) Quantitative analyses of platelet desialylation showed that RCA-I (D), ECL (E) or sWGA (F) FITC-labeled lectins increased significantly in septic patients with thrombocytopenia (n = 127) compared to those without thrombocytopenia (n = 134). RCA-I, ECL or sWGA: Mean ± SD. *P* values between 3 groups were obtained by one-way ANOVA. **P* < 0.05. ****P* < 0.001.


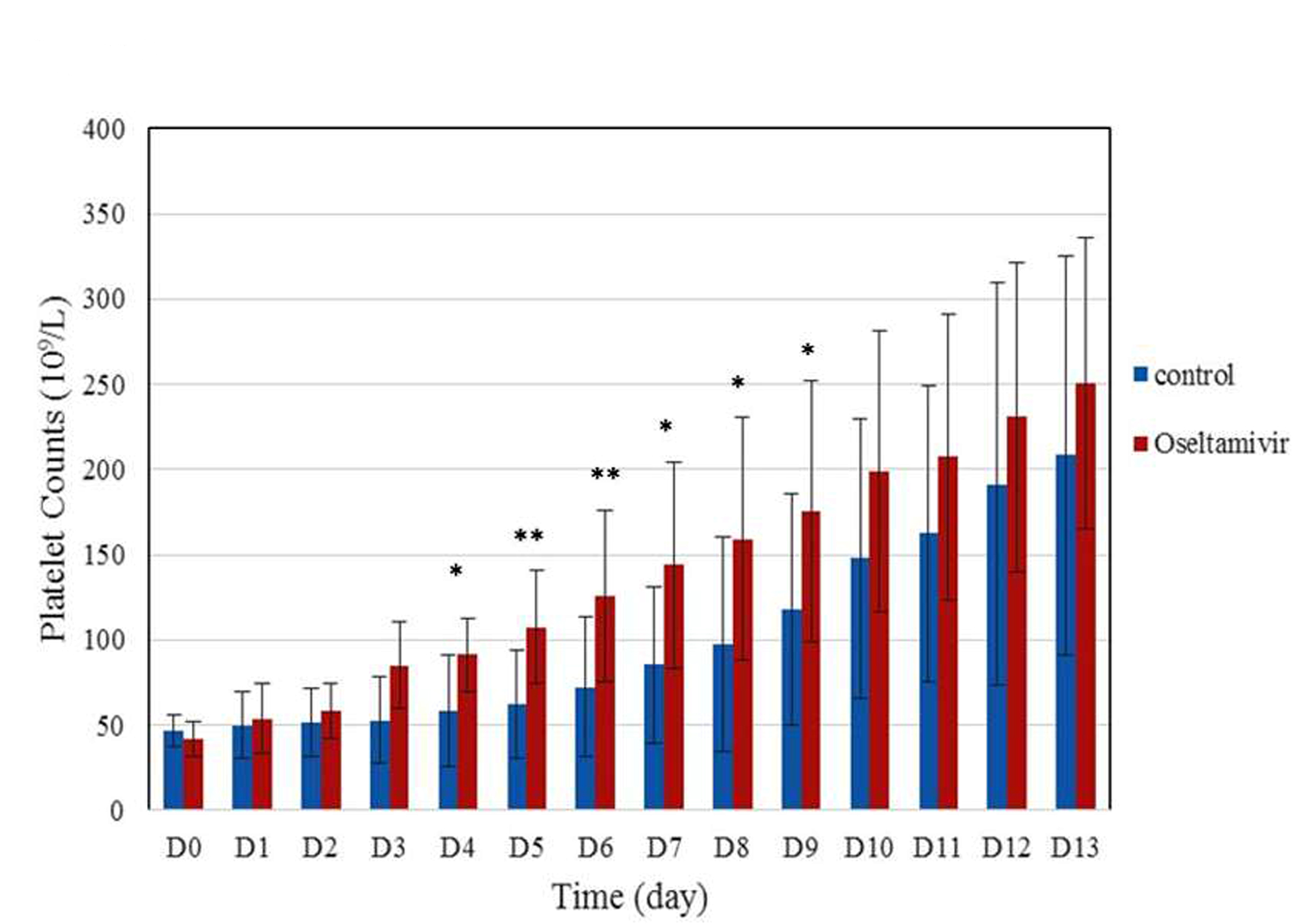


**Figure S2 Changes in platelet counts of the oseltamivir and control groups over time.** A statistically significant difference between the oseltamivir group (n = 54) and the control group (n = 52) was exhibited from the fourth through ninth day after the initiation of oseltamivir treatment. Analysis of platelet counts between the oseltamivir group and the control group applied non-parameter statistical analysis, but with a mean ± standard deviation mapping. *P* values were obtained by 2-tailed Student’s *t*-tests. ^*^*P* < 0.05. ^**^*P* < 0.01.

**
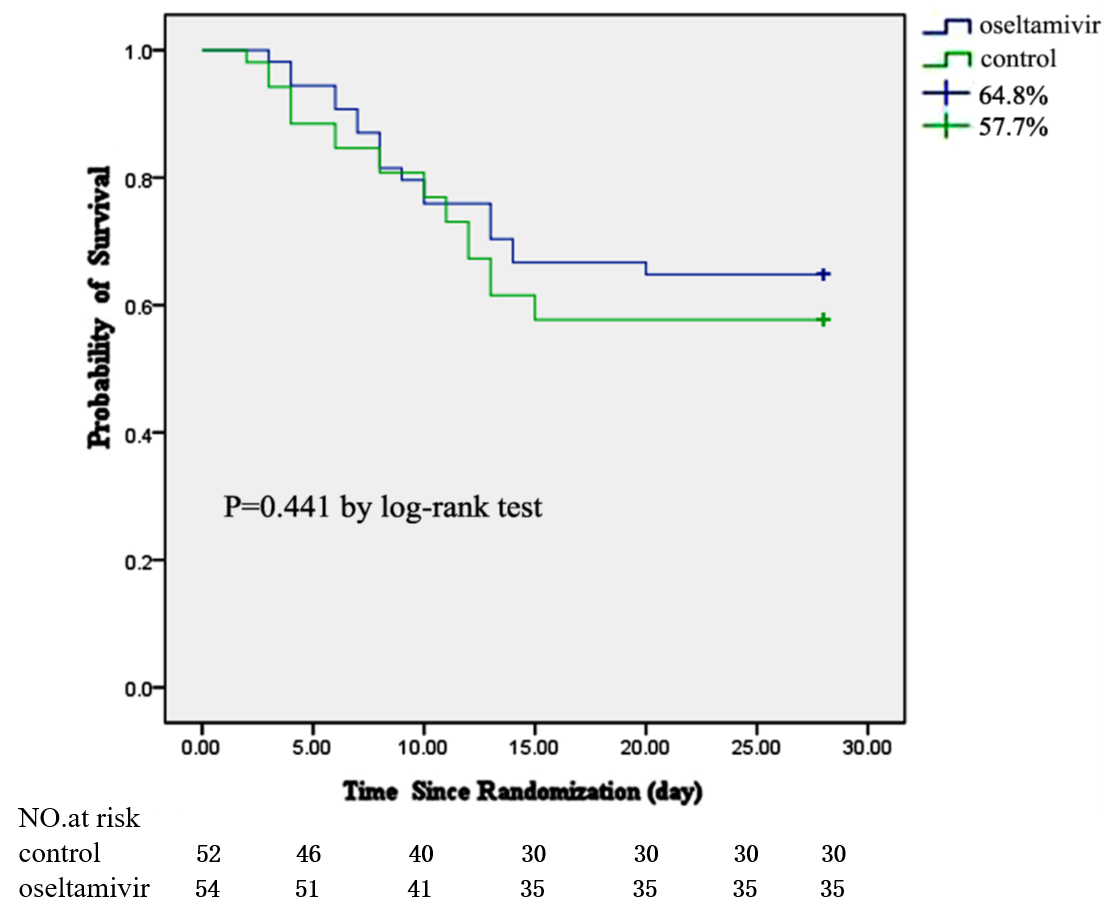
**

**Figure S3. Survival curves of thrombocytopenia patients, according to treatment group.** Survival analysis was conducted based on whether oseltamivir was used, and the difference in survival over time between the oseltamivir group and the control group was compared using the log-rank test.


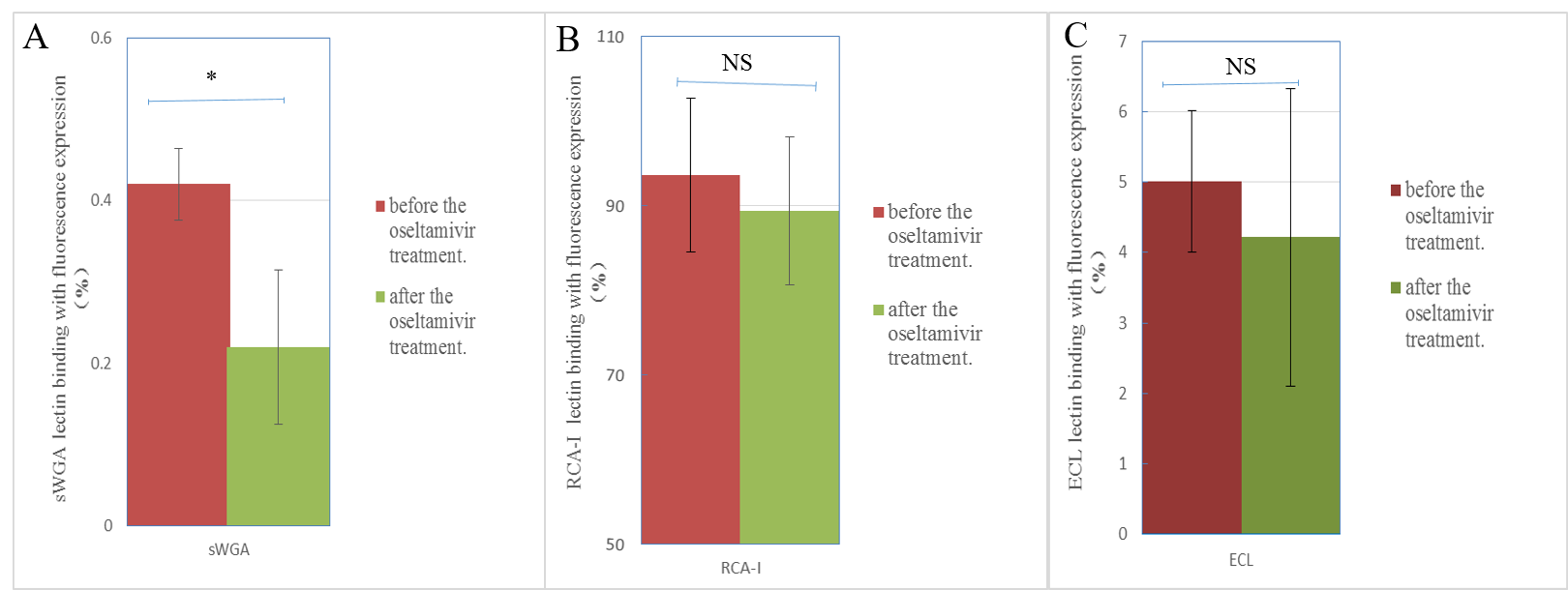


**Figure S4. Platelet desialylation levels before and after the oseltamivir treatment.** Quantitative analyses of platelet desialylation showed that sWGA (A) FITC-labeled lectins declined significantly 14 days after the initiation of oseltamivir treatment compared to those before treatment in sever septic patients with thrombocytopenia (n = 25). However, neither RCA-I (B) nor ECL (C) FITC-labeled lectins showed significant change after oseltamivir treatment. RCA-I, ECL or sWGA: Mean ± SD. *P* values were obtained by 2-tailed Student’s *t*-tests. **P* < 0.05.

**Tables**

**Table S1. Baseline characteristics of the all patients (n = 261)**

| **Parameters** | **No. (%) or Median (IQR)** |
| --- | --- |
| Age (yr) | 68 (55, 77.5) |
| Male | 175 (67.1) |
| APACHE II | 19.5 (11.3, 24) |
| Predicted death rate (%) | 32.24 (20.8, 49.3) |
| SOFA | 7 (4.25, 11） |
| PCT (ng/ml) | 3.3 (0.55, 11.24） |
| Platelet count (× 10^9^/L) | 72 (40, 149） |
| Medical history |  |
| Hypertension | 88 (34.6） |
| Diabetes mellitus | 47 (18.5） |
| Cardiovascular disease | 77 (30.3） |
| Neurologic disease | 44 (17.3） |
| Chronic renal failure | 6 (2.3） |
| Primary reason for admission | |
| Respiratory | 85 (32.6) |
| Postoperative | 31 (11.9) |
| Nervous | 9 (3.40) |
| Sever Sepsis | 41 (15.7) |
| Infection shock | 19 (7.28) |
| CRRT | 12 (4.60) |
| Virus Infection | 53 (20.3) |
| Others | 11 (4.21) |
| Infection pathogens (%) |  |
| Escherichia coli | 37 ( 14.2) |
| Klebsiella pneumoniae | 33 (12.5) |
| Pseudomonas aeruginosa | 40 (15.3) |
| Acinetobacter Baumanii | 20 (7.7) |
| Staphylococcus aureus | 26 (10.0) |
| Streptococcus pneumoniae | 3 (1.15) |
| [Enterococcus](javascript:void(0);) [faecium](javascript:void(0);) | 9 (3.45) |
| [Proteus](javascript:void(0);) [mirabilis](javascript:void(0);) | 1 (0.38) |
| Enterococcus gallinarum | 3 (1.15) |
| Aspergillus | 10 (3.83) |
| Candida Albicans | 3 (1.15) |
| Viruses | 53 (20.3) |
| New Bunia virus | 21 (8.05) |
| Influenza A virus subtype H1N1 | 12 (4.60) |
| Others | 20 (7.66) |
| Infection sites(%) |  |
| Lung | 98 (37.5) |
| Abdominal cavity | 26 (9.96) |
| Bloodstream | 41 (15.7) |
| Hepatobiliary system | 17 (6.51) |
| Urinary system | 14 (5.36) |
| Surgical incision | 6 (2.30) |
| Skin and soft tissue | 4 (1.53) |
| Central nervous system | 3 (1.15) |
| Others | 52 (19.9) |
| Platelet desialylation level (%) | |
| RCA-I | 95.6 (89.8, 99.2) |
| ECL | 3.45 (2.3, 5.6) |
| sWGA | 0.32 (0.2, 0.5) |

Data are presented as number (percentage) or median (Interquartile range). IQR, interquartile range; APACHEII: acute physiology and chronic health evaluation scoring system; SOFA: sequential organ failure assessment; ICU: intensive care unit; CRRT: continuous renal replacement therapy; PCT: procalcition; RCA-I: *Ricinus communis* agglutinin I; ECL: *Erythrina cristagalli* lectin; sWGA: Succinyl *Triticum vulgare* lectin.

**Table S2. Factors affecting the thrombocytopenia in the multiple stepwise regression model (n = 261)**

| **Variables** | **Standardized ß** | **t** | ***P* value** |
| --- | --- | --- | --- |
| SOFA | -0.313 | -5.429 | 0.001 |
| Bloodstream infection | -0.134 | -2.326 | 0.021 |
| New Bunya virus infection | -0.219 | -3.912 | 0.000 |
| Pseudomonas eruginosa infection | 0.136 | 2.426 | 0.016 |
| Staphylococcus aureus infection | 0.125 | 2.221 | 0.027 |

SOFA: sequential organ failure assessment.

**Table S3. Multivariate analysis of the effects of various clinical parameters at study entry on the odds ratio for 28-day mortality (n = 261)**

| **Variables** | **ß** | **OR** | **95% CI** | ***P* value** |
| --- | --- | --- | --- | --- |
| Age (yr) | -0.009 | 0.991 | 0.946-1.037 | 0.678 |
| Male | -0.233 | 0.792 | 0.136-4.68 | 0.792 |
| SOFA | 0.592 | 1.808 | 1.246-2.623 | 0.002 |
| Platelet count | -0.038 | 0.963 | 0.930-0.997 | 0.033 |
| Mechanical ventilation | 0.992 | 2.698 | 0.353-20.629 | 0.339 |
| CRRT | -1.912 | 0.148 | 0.014-1.531 | 0.109 |
| ARDS | 0.461 | 1.586 | 0.213-11.834 | 0.653 |
| Septic shock | -1.352 | 0.259 | 0.030-2.233 | 0.219 |

SOFA, sequential organ failure assessment; ARDS, ] acute respiratory distress syndrome; CRRT, continuous renal replacement therapy.

**Table S4. Baseline demographic data of patients treated with antibiotics plus oseltamivir (oseltamivir group) or with antibiotics alone (control group**)

| **Variables** | **Control**  (**n = 52)** | **Oseltamivir**  (**n = 54)** | ***P* value** |
| --- | --- | --- | --- |
| Age (yr) | 67 (55, 75.8) | 71.5 (51.8, 79.3) | 0.180 |
| Sex (male) | 34 (65.4) | 37 (68.5) | 0.837 |
| APACHE II | 18.9 ± 7.25 | 17.6 ± 7.22 | 0.368 |
| Predicted death rate (%) | 34.6 ± 20.6 | 37.6 ± 21.2 | 0.459 |
| SOFA | 10 (5, 12) | 9.5 (5, 12.3) | 0.443 |
| Platelet count (× 10^9^/L) | 43.15 ± 19.4 | 45.4 ± 21.8 | 0.579 |
| PCT (ng/ml) | 6.68 (0.542, 34.1) | 6.69 (0.65, 42.8) | 0.307 |
| Medical history |  |  |  |
| Hypertension | 16 (30.8) | 14 (25.9) | 0.151 |
| Diabetes mellitus | 14 (26.9) | 12 (22.2) | 0.153 |
| Neurologic disease | 6 (11.5) | 5 (9.3) | 0.230 |
| Cardiovascular disease | 14 (26.9) | 13 (24.1) | 0.150 |
| Chronic renal failure | 2 (3.85) | 3 (5.56) | 0.320 |
| Mean arterial pressure (mmHg) | 81.7 ± 13.3 | 78.9 ± 16.1 | 0.352 |
| Heart rate (beat/min) | 92.5 (80, 114) | 100 (84, 114) | 0.234 |
| White blood cell ( × 10^9^/L) | 9.48 ± 7.41 | 12.5 ± 9.41 | 0.074 |
| PT (s) | 14.0 (12.1, 16.1) | 13.5 (12.2, 18.5) | 0.723 |
| APTT (s) | 40. 1 ± 10.0 | 43.0 ± 14.6 | 0.210 |
| HCT (%) | 30.8 (27.9, 36.7) | 30.7(28.1, 37.9) | 0.867 |
| BUN (mmol/L) | 8.10 (4.01, 14.6) | 8.35 (4.59, 20.1) | 0.203 |
| Creatinine (μmol/L) | 110.5 (63.7, 250) | 118 (70, 326) | 0.491 |
| Total bilirubin (μmol/L) | 17.45 (11.4, 29.8) | 20.7 (12.2, 36.8) | 0.435 |
| Albumin (g/L) | 23.8 ± 6.83 | 25. 1 ± 4.67 | 0.229 |
| Mechanical ventilation | 33 (61.5) | 30 (61.9) | 0.119 |
| CRRT | 12 (23.1) | 15 (47.6) | 0.152 |
| ARDS | 13 (26.9) | 15 (27.8) | 0.175 |
| Infection pathogens (%) |  |  |  |
| Escherichia coli | 15(28.8) | 10 (18.5) | 0.256 |
| Klebsiella pneumoniae | 3 (5.77) | 6 (11.1) | 0.489 |
| Pseudomonas aeruginosa | 5 (9.61) | 2 (3.70) | 0.266 |
| Staphylococcus aureus | 2 (3.85) | 3 (5.56) | 0.999 |
| Streptococcus pneumonae | 2 (3.85) | 0 (0.00) | 0.238 |
| Enterococcus faecium | 2 (3.85) | 2 (3.70) | 0.899 |
| Acinetobacter Baumanii | 2 (3.85) | 4 (7.41) | 0.679 |
| Aspergillus | 1 (1.92) | 3 (5.61) | 0.618 |
| New Bunia virus | 9 (17.3) | 12 (22.2) | 0.628 |
| Influenza A virus subtype H1N1 | 1 (1.92) | 4 (7.41) | 0.363 |
| Others | 10 (19.2) | 8 (14.8) | 0.611 |
| Infection sites (%) |  |  |  |
| Lung | 15 (28.8) | 12 (22.2) | 0.506 |
| Bloodstream | 10 (19.2) | 16 (29.6) | 0.262 |
| Abdominal cavity | 10 (19.2) | 5 (9.3) | 0.170 |
| Hepatobiliary system | 5 (9.61) | 3 (5.61) | 0.484 |
| Urinary system | 4 (7.69) | 4 (7.41) | 0.999 |
| Others | 8 (15.4) | 14 (25.9) | 0.233 |
| Duration of antimicrobial treatment (day) | 11.0 (9.0, 13.25) | 10.0 (9.0, 13.0) | 0.418 |

APACHE II, acute physiology and chronic health evaluation scoring system; SOFA, sequential organ failure assessment; PCT, procalcitonin; ARDS, acute respiratory distress syndrome; MAP, mean arterial pressure; HCT, red blood cell specific volume; PT, prothrombin time; APTT, activated partial thromboplastin time; SA, sialic acid; CRRT, continuous renal replacement therapy; BUN, blood urea nitrogen.

**Table S5. The adverse events in oseltamivir treatment group and control group in study II (n = 106)**

| **Side effects** | **Control**  **(n = 52)** | **Oseltamivir**  **(n = 54)** | ***P* value** |
| --- | --- | --- | --- |
| nausea | 5 (9.62%) | 7 (12.9%) | 0.21 |
| vomit | 3 (5.77%) | 5 (9.26%) | 0.23 |
| [bronchitis](../../../../C:/Users/Administrator/AppData/Local/Youdao/Dict/Application/6.3.69.8341/resultui/frame/javascript:void(0)%3B) | 3 (5.77%) | 2 (3.70%) | 0.31 |
| i[nsomnia](../../../../C:/Users/Administrator/AppData/Local/Youdao/Dict/Application/6.3.69.8341/resultui/frame/javascript:void(0)%3B) | 2 (3.85%) | 2 (3.70%) | 0.38 |
| vertigo | 1 (1.92%) | 1 (185%) | 0.50 |
